# Actual and predicted prevalence of alcohol consumption during pregnancy in Latin America and the Caribbean: systematic literature review and meta-analysis

**DOI:** 10.26633/RPSP.2017.89

**Published:** 2017-06-19

**Authors:** Shannon Lange, Charlotte Probst, Navrose Heer, Michael Roerecke, Jürgen Rehm, Maristela G. Monteiro, Kevin Shield, Claire de Oliveira, Svetlana Popova

**Affiliations:** 1 Institute for Mental Health Policy Research Center for Addiction and Mental Health Ontario Canada Institute for Mental Health Policy Research, Center for Addiction and Mental Health, Toronto, Ontario, Canada.; 2 Pan American Health Organization Pan American Health Organization Washington DC. United States of America Pan American Health Organization, Washington DC, United States of America.

**Keywords:** Alcohol drinking, pregnancy, prenatal care, fetal development, Latin America, Caribbean Region, Consumo de bebidas alcohólicas, embarazo, atención prenatal, desarrollo fetal, América Latina, Región del Caribe, Consumo de bebidas alcoólicas, gravidez, cuidado pré-natal, desenvolvimento fetal, América Latina, Região do Caribe

## Abstract

**Objective:**

*To estimate the prevalence of alcohol consumption during pregnancy among the general population of Latin America and the Caribbean, by country, in 2012*.

**Methods:**

*Three steps were taken: a comprehensive, systematic literature search; meta-analyses, assuming a random-effects model for countries with published studies; and regression modelling (data prediction) for countries with either no published studies or too few to obtain an estimate*.

**Results:**

*Based on 24 existing studies, the pooled prevalence of alcohol consumption during pregnancy among the general population was estimated for Brazil (15.2%; 95% confidence interval [95%CI]: 10.4%–20.8%) and Mexico (1.2%; 95%CI: 0.0%–2.7%). The prevalence of alcohol consumption during pregnancy among the general population was predicted for 31 countries and ranged from 4.8% (95%CI: 4.2%–5.4%) in Cuba to 23.3% (95%CI: 20.1%–26.5%) in Grenada*.

**Conclusions:**

*Greater prevention efforts and measures are needed in the countries of Latin America and the Caribbean to prevent pregnant women from consuming alcohol during pregnancy and decrease the rates of Fetal Alcohol Spectrum Disorder. Additional high quality studies on the prevalence of alcohol consumption during pregnancy in Latin America and the Caribbean are also needed*.

## RESUMEN

**Objetivo.:**

Calcular la prevalencia del consumo de alcohol durante el embarazo en la población general de América Latina y el Caribe desglosada por país y para el año 2012.

**Métodos.:**

Se siguieron tres pasos: 1) una búsqueda bibliográfica sistemática y amplia; 2) metanálisis, en los que se supuso un modelo de efectos aleatorios respecto de los países sobre los que se disponía de estudios publicados; y 3) el modelado de regresión (predicción de datos) respecto de los países sobre los que no se disponía de ningún estudio publicado o estos eran demasiado escasos para obtener estimaciones.

**Resultados.:**

Sobre la base de los 24 estudios existentes, se calculó la prevalencia combinada del consumo de alcohol durante el embarazo en la población general de Brasil (15,2 %; intervalo de confianza [IC] del 95 %: 10,4-20,8 %) y México (1,2 %; IC del 95 %: 0,0-2,7 %). También se calculó la prevalencia prevista del consumo de alcohol durante el embarazo en la población general de 31 países, cálculo que arrojó datos comprendidos entre 4,8 % (IC del 95 %: 4,2-5,4 %) en Cuba y 23,3 % (IC del 95%: 20,1-26,5 %) en Granada.

**Conclusiones.:**

Es preciso redoblar los esfuerzos de prevención e intensificar las medidas en los países de América Latina y el Caribe para impedir que las embarazadas consuman alcohol durante el embarazo a fin de reducir las tasas de los trastornos del espectro alcohólico fetal. También se deben realizar más estudios de buena calidad sobre la prevalencia del consumo de alcohol durante el embarazo en América Latina y el Caribe.

**Objetivo.:**

Estimar a prevalência do consumo de álcool na gravidez na população geral da América Latina e Caribe, por país, em 2012.

**Métodos.:**

Três etapas foram realizadas: (i) uma busca sistemática abrangente da literatura científica, (ii) meta-análises a partir de um modelo de efeitos aleatórios para os países com estudos publicados e (iii) modelos de regressão (predição de dados) para os países sem nenhum estudo publicado ou com um número muito pequeno de estudos para obter uma estimativa.

**Resultados.:**

Com base em 24 estudos identificados, estimou-se a prevalência conjunta do consumo de álcool na gravidez na população geral no Brasil (15,2%; intervalo de confiança de 95% [IC 95%] 10,4%–20,8%) e no México (1,2%; IC 95% 0,0%–2,7%). A prevalência do consumo de álcool na gravidez na população geral foi prevista em 31 países, variando de 4,8% (IC 95% 4,2%–5,4%) em Cuba a 23,3% (IC 95% 20,1%–26,5%) em Granada.

**Conclusões.:**

Um grande esforço de prevenção com a adoção de medidas preventivas se faz necessário nos países da América Latina e Caribe para prevenir o uso de álcool entre gestantes durante a gravidez e reduzir os índices de transtornos do espectro alcoólico fetal. Também são necessários outros estudos de alta qualidade da prevalência do consumo de álcool na gravidez na América Latina e no Caribe.

Research on alcohol consumption among women in the Region of the Americas has primarily focused on Canada and the United States of America, and less on Central and South America and the Caribbean. Nevertheless, alcohol consumption among women in these areas remains problematically high compared to the world average. According to the *Global Status Report on Alcohol and Health* (1), in 2010 the highest per capita consumption of pure alcohol among women (defined as 15+ years of age) in Central America was in Panama (4.7L), Costa Rica (3.2L), and Mexico (2.6L); in South America, Chile (5.5L), Argentina (5.2L), and Paraguay (5.2L); and in the Caribbean, Grenada (7.3L), Saint Lucia (5.9L), and Saint Kitts and Nevis (4.7L). Notably, in the same year, women in most of the countries (78%) in Latin America and the Caribbean (LAC) had higher pure alcohol per capita consumption (APC) than the global average APC for women (2.7L) (1). Furthermore, in 2010 the highest prevalence rates of heavy episodic drinking (≥ 60mL of pure alcohol on at least one occasion monthly) among women in Central America were in Guatemala (14.2%), El Salvador (13.6%), and Nicaragua (6.6%); in South America, Paraguay (41.0%), Venezuela (21.8%), and Brazil (11.1%); and in the Caribbean, Saint Kitts and Nevis (33.1%), Dominica (29.6%), and Trinidad and Tobago (24.4%) (1). The majority of these figures are significantly higher than the global prevalence of heavy episodic drinking among women drinkers, which was 5.7% in 2010 (1).

These data demonstrate that not only are women in LAC drinking at high rates, but many of them are engaging in risky drinking patterns. As such, it is likely that some women may continue to drink during pregnancy or before becoming aware of a pregnancy. Furthermore, a recent study found LAC to have the highest proportion of unintended pregnancies (56%), while other regions, i.e., Africa, Asia, Europe, North America, and Oceania, ranged from 35% to 51%; the worldwide average being 40% (2). Coupled with the relatively high rates of alcohol consumption and risky drinking patterns, this may lead to an increased risk of alcohol-exposed pregnancies in these countries.

It is a well-known fact that alcohol is a teratogen that can cause significant harm to a developing fetus. Worldwide, the majority of clinical guidelines advocate that women who are pregnant or planning to become pregnant should abstain from alcohol due to potential adverse health consequences, which could include spontaneous abortion, stillbirth, intrauterine growth retardation, low birth weight, preterm labor, birth defects, and Fetal Alcohol Spectrum Disorder (FASD) (3–8). FASD encompasses a broad array of physical defects and cognitive, behavioral, emotional, and adaptive functioning deficits, as well as congenital anomalies. The effects of alcohol on the fetus are permanent, and as such, many people with FASD require life-long support, resulting in a significant cost to society (9, 10).

There is a general paucity of research on the prevalence of FASD in LAC countries. Few studies have reported the prevalence of Fetal Alcohol Syndrome (FAS; the most severe and visibly identifiable form of FASD) in South America. Specifically, it has been reported to be 0.1% among the general population of Brazil and Uruguay (11, 12). However, the prevalence of FAS and partial FAS among children in care in Chile has been reported to be much higher: 15% among those in foster homes (13), and 16% among those in child welfare custody and homes for those with mental deficiencies (14). A study of 103 children living in the United States who were adopted from Guatemala found that 28% had the phenotypic facial features suggestive of prenatal alcohol exposure (15).

The objective of the current study was to estimate the prevalence of alcohol consumption (any amount) during pregnancy among the general population of LAC.

## MATERIALS AND METHODS

In order to estimate the prevalence of alcohol consumption (any amount) during pregnancy among the general population of LAC,^3^ three steps were taken: (i) a comprehensive systematic literature search; (ii) meta-analyses based on the existing studies; and (iii) regression modelling (data prediction) for countries with either no published studies or too few to obtain an estimate.

### Comprehensive systematic literature search

#### Search strategy.

A comprehensive systematic literature review was conducted and reported according to the standards set out in *Preferred Reporting Items for Systematic Reviews and Meta-Analyses* (16), and guided by the overview of Egger and colleagues (17). The search was performed to identify studies published in January 1984 – June 2014, i.e., in the last 30 years, without language restriction. The search was conducted in multiple electronic bibliographic databases: MEDLINE, PubMed, EMBASE, Web of Science (including Science Citation Index, Social Sciences Citation Index, Arts and Humanities Citation Index), PsycINFO, ERIC, Ebscohost, CINAHL, Campbell Collaboration, the Cochrane Database of Systematic Reviews, CSA Sociological Abstracts, Social Work Abstracts, Canadian Centre on Substance Abuse Library Collection Database, National Institute on Alcohol Abuse and Alcoholism’s Alcohol and Alcohol Problems Science Database (ETOH), Scopus, Centre for Addiction and Mental Health Library Database, and Google Scholar. Multiple combinations of the following keywords were used: alcohol, binge, ethanol; behavi*, consum*, drink*; maternal, mother, primigravida, wom*n; pregnant, pregnanc*, prenatal; epidemiology, frequenc*, occurrence, prevalence; and Antigua and Barbuda, Argentina, Bahamas, Barbados, Belize, Bolivia, Brazil, Caribbean, Central America, Chile, Colombia, Costa Rica, Cuba, Dominica, Dominican Republic, Ecuador, El Salvador, Grenada, Guatemala, Guyana, Haiti, Honduras, Jamaica, Latin America, Mexico, Nicaragua, Panama, Paraguay, Peru, Saint Kitts and Nevis, Saint Lucia, Saint Vincent and the Grenadines, South America, Suriname, Trinidad and Tobago, Uruguay, and Venezuela. In addition, the content pages of the major epidemiological journals, as well as citations in the relevant articles, were manually screened.

#### Study selection.

Study selection began by screening the titles and abstracts of studies for inclusion. Then, full-text articles of all studies screened as potentially relevant were considered. The following inclusion criteria were then applied to determine eligibility: (i) consisted of original quantitative research published in a peer-reviewed journal or scholarly report (i.e., a report written by scholars/professionals who are experts in the field of alcohol use during pregnancy and published on an institutional/government website); and (ii) measured the prevalence of alcohol consumption during pregnancy among the general population in any country in Latin America or the Caribbean. Articles were excluded if they (i) excluded abstainers from their samples (which would lead to an inflated estimate), or (ii) reported a pooled estimate of alcohol use during pregnancy by combining several studies. Regarding the latter exclusion criterion, the primary studies were included. Two investigators conducted each study selection step independently; any disagreements were reconciled by team discussion. In cases where multiple studies used the same dataset or cohort, the study with the larger sample size was included.

#### Data extraction.

All data were extracted by one investigator, and then independently crosschecked by a second investigator for accuracy against the original studies. The following variables were extracted: country; study year(s); timing of data collection; sample size; setting; sociodemographic status, i.e., income, education, occupation/employment status, marital status; instrument used to obtain alcohol use data; percentage of women who consumed alcohol, binge drank, used drugs, and/or smoked during pregnancy; as well as the number of previous pregnancies, number of previous live births, and percentage of unplanned pregnancies (for the index pregnancy). Using a checklist for observational studies developed *a priori* based on the criteria described and validated in Wong and colleagues (18), two investigators independently appraised the quality of each study. The following criteria were used: (i) representativeness of the sample (i.e., used probability or non-probability sampling); (ii) adequate sample size (≥ 300 participants); (iii) utilization of a validated tool to ascertain alcohol use during pregnancy, e.g., the Alcohol Use Disorders Identification Test–Consumption (AUDIT-C; 19), CRAFFT screening interview (20), and 4P’s Plus screen (21); (iv) adequate response/participation rate (≥ 60%), and (v) whether or not the study subjects were described. All discrepancies in quality ratings were reconciled by team discussion. Inter-rater agreement for inclusion and quality assessment was assessed using Fleiss’s kappa statistics (22). Training of coders to achieve sufficient (> 0.80) inter-rater reliability was conducted.

### Meta-analyses

A meta-analysis was performed for each country where at least three estimates of the prevalence of alcohol consumption during pregnancy were available. The criterion of three estimates was chosen in order to reduce the chance of presenting biased estimates that were not generalizable. In cases where a study reported the prevalence of alcohol consumption during pregnancy by (i) the time at which alcohol consumption was measured, or (ii) the trimester of alcohol consumption, (iii) awareness of pregnancy (i.e., before and after pregnancy recognition), or by (iv) method of ascertainment (e.g., medical chart and questionnaire), preference was given to studies that: (i) assessed alcohol consumption soon after delivery (within 6 weeks postpartum), (ii) reported the prevalence of alcohol consumption during the course of the entire pregnancy (as opposed to just in the first trimester, for example), (iii) assessed alcohol consumption after pregnancy recognition, and (iv) used a validated tool for ascertainment of alcohol consumption.

The prevalence estimates underwent a double arcsine transformation so that the data followed a normal distribution, an assumption needed when statistically combining estimates (23). In each case, in order to combine prevalence estimates, the meta-analysis was conducted using a random-effects model (24). Heterogeneity between double arcsine-transformed estimates of drinking during pregnancy was assessed using the I^2^ statistic (25). Publication bias was examined by visually inspecting the funnel plot (standard error plotted against the point estimate) for a skewed distribution and by employing Egger’s regression test for small-study effects (26).

### Data prediction

For the countries with either no published data or fewer than three published studies on the topic, prevalence estimates were predicted using prevalence data of more than 300 studies from 50 countries (i.e., data were not limited to the Americas; 27). A fractional response regression model was employed to restrict predictions to values between 0 and 1 (28). Country- and year-specific explanatory variables were gross domestic product (adjusted for purchasing power parity) per capita (29), mean total consumption of alcohol among women (1), and the WHO Region within which the country was located. If study year(s) was not reported, the year of publication was used. If the study was conducted across 2 or more years, a value for each of the explanatory variables was calculated as the average of the value from the study’s start year to that of its end year. Total mean alcohol consumption among women was estimated as the proportion of the total amount of alcohol consumed in the respective country that was consumed by women in 2010/2012 (1). In order to account for the heterogeneity within the Region of the Americas and the European Region, particularly with respect to income level and drinking culture, these regions were divided by income level (for Europe: countries that belong to the European Union versus countries that do not; and for the Americas: Canada and the United States versus the remaining countries).

Predictions were based on the values of the above named explanatory variables for the year 2012. In order to be conservative, the confidence interval (CI) for each estimate was based on the standard deviation of the prediction. All statistics were performed using R version 3.1.0 (R Development Core Team, Vienna, Austria).

## RESULTS

### Comprehensive systematic literature search

Initially, the search yielded a total of 956 publications for LAC countries; 954 articles identified through the electronic search, and two through the manual search. After removing 542 duplicate articles, the remaining 414 articles were screened using titles and abstracts. The full-texts of 87 articles were retrieved for further consideration, 63 of which were subsequently excluded. A total of 24 articles (all from peer-reviewed journals) contained relevant data and were retained for data extraction. Inter-rater agreement for inclusion and quality assessment was excellent (κ = 0.96 and κ = 0.93, respectively). Data from published studies on the prevalence of alcohol consumption during pregnancy were available from 5 of the 33 countries in LAC (Brazil, *n* = 17 (30–46); Chile, *n* = 2 (47, 48); Guatemala, *n* = 1 (49); Mexico, *n* = 3 (50–52); and Uruguay, *n* = 1 (53); there were no studies of the Caribbean).

All of the included studies presented data on self-reported alcohol consumption during pregnancy. The characteristics of the participants (e.g., sociodemographic characteristics) in each study are available from the authors upon request.

In regard to study quality, 88% used non-probability sampling and only 13% utilized a validated tool to ascertain alcohol use. However, 96% of the studies described their study participants, 92% had an adequate sample size, and 54% had a reasonable participation rate (quality ratings of the included studies are available from the authors upon request).

### Prevalence reported by individual studies

Overall, the prevalence of alcohol consumption during pregnancy among the general population in the 24 identified studies ranged from 0.4% in Mexico (50) to 57.4% in Chile (47; Table 1). Only three studies reported the prevalence of binge/excessive drinking of women during pregnancy in the general population: 5.1% binge drank (≥ 5 drinks on a single occasion) in Brazil (34), 0.9% had “excessive consumption” (not further defined) in Brazil (41), and 1.0% consumed an average of four drinks per drinking day in Chile (47).

**TABLE 1:** Study characteristics and prevalence of alcohol consumption (any amount) and binge drinking during pregnancy among the general population reported in the identified studies, Latin America and the Caribbean, **2004–2013**

Country (city/province)	Reference	Time period	Timing of data collection	Sample size	Data collection instrument	Women who consumed alcohol during pregnancy		Women who “binge drank” during pregnancy	
						n	%	n	%
Central America									
Guatemala (Guatemala City)	Johri et al., 2011 (49)	2006	During pregnancy	1 897	Questionnaire	93	4.9	NA^a^	NA
Mexico (Baja California, Tijuana)	Castro-Espinoza et al., 2009 (50)	2006–2007	Retrospective (post-partum)	730	Questionnaire	3	0.4	NA	NA
Mexico (Mexico City)	Doubova et al., 2007 (51)	2003–2004	During pregnancy (mean: 29.1 weeks)	386	Questionnaire	5	1.3	NA	NA
Mexico (Jalisco, Guadalajara)	Peña & Matute, 2010 (52)	1991–1998	Retrospective	78 871	Medical chart	1 909	2.4	NA	NA
South America									
Brazil (Sergipe, Aracaju)	Almeida et al., 2010 (30)	2005	Retrospective (post-partum)	4 712	Questionnaire	977	20.7	NA	NA
Brazil (Rio Grande do Sul, Porto Alegre)	Buss et al., 2009 (31)	2006–2007	During pregnancy (16-36 weeks)	578	Questionnaire	97	16.8	NA	NA
Brazil (Rio Grande do Sul)	Cesar et al., 2009 (32)	2007	During pregnancy	2 523	Questionnaire	96	3.8	NA	NA
Brazil (Rio Grande do Sul, Pelotas)	da Silva et al., 2010 (33)	2006–2008	During pregnancy (> 14 weeks; mean: 27.7)	1 204	CAGE^c^	99	8.2	NA	NA
Brazil (Minas Gerais)	De Souza et al., 2012 (34)	2009	Retrospective (12-24 hours [hrs]) post-partum)	493	AUDIT^b^	114 2-4 times/month: 49 2-3 times/week: 18 Almost daily: 7 On a typical occasion: 5-6 drinks: 10 7-9 drinks: 5 10+ drinks: 10	23.1 2-4 times/month: 9.9 2-3 times/week: 3.7 Almost daily: 1.4 On a typical occasion: 5-6 drinks: 2.0 7-9 drinks: 1.0 10+ drinks: 2.0	≥ 1 binge drinking episode (≥ 5 drinks/occasion): 25	≥ 1 binge drinking episode: 5.1
Brazil (Rio de Janeiro)	Freire et al., 2009 (35)	1999–2006	During pregnancy	433	Questionnaire	107	24.4	NA	NA
Brazil (Fortaleza, Manaus, Porto Alegre, Rio de Janeiro, and Salvador)	Kroeff et al., 2004 (36)	1991–1995	During pregnancy (21-28 weeks)	5 539	Questionnaire	483	17.5	NA	NA
Brazil (Bahia, Feira de Santana)	Lopes Brito et al., 2011 (37)	2009	Retrospective (4-5 years)	438	T-ACE^c^	44	7.9	NA	NA
Brazil (Bahia)	Mello et al., 2014 (38)	2008–2010	During pregnancy	2 761	Questionnaire	218	40.6	NA	NA
Brazil (Pernambuco, Recife, São Paulo, Campinas)	Melo et al., 2011 (39)	NA	Retrospective (post-partum)	555	Questionnaire	99	22.0	NA	NA
Brazil (Rio de Janeiro)	Moraes & Reichenheim, 2007 (40)	2000	Retrospective (48 hrs post-partum)	537	CAGE, T-ACE, TWEAK^c^	218	40.6	NA	NA
Brazil (São Paulo, Ribeirão Preto)	Pinheiro et al., 2005 (41)	2001	During third trimester of pregnancy	450	Questionnaire	99 Drank daily: 5	22.0 Drank daily: 1.1	Excessive consumption: 4	Excessive consumption: 0.9
Brazil (Rio Grande do Sul, Pelotas)	Santos et al., 2005 (42)	1993	Retrospective (24 hrs post-partum)	5 189	Questionnaire	Drank at least once/month: 259	Drank at least once/month: 5.0	NA	NA
Brazil (Juiz de Fora, Minas Gerais)	Silva et al., 2010 (43)	2006–2008	During pregnancy (20-42 weeks; mean: 33 [SD = 4.4])	260	AUDIT	64	24.6	NA	NA
Brazil (Rio de Janeiro)	Viellas et al., 2013 (44)	2000–2001	Retrospective (post-partum)	8 961	Questionnaire/medical record	1 656	18.5	NA	NA
Brazil (São Paulo)	Vogt et al., 2012 (45)	2004–2006	During pregnancy (≤ 16-32 weeks)	334	Questionnaire	23	6.9	NA	NA
Brazil (Bahia, Santo Amaro)	Zentner et al., 2008 (46)	2002	Retrospective (upon being admitted for delivery)	55	Questionnaire	1	1.8	NA	NA
Chile (Santiago)	Aros et al., 2006 (47)	1995–2000	During pregnancy (mean: 16.7 weeks)	9 628	Questionnaire	5 524 Drank < 12g/month: 2 847 Drank > 12g/month, but < 12g/day: 2 323 Drank ≥ 12 g/day: 354	57.4 Drank < 12g/month: 29.6 Drank > 12g/month, but < 12 g/day: 24.1 Drank ≥ 12g/day: 3.7	Consumed an average of 4 drinks/day: 101	Consumed an average of 4 drinks/day: 1.0
Chile (Valvida)	Barria et al., 2008 (48)	2005–2006	Retrospective (48 hrs post-partum)	315	Questionnaire	50	15.9	NA	NA
Uruguay	Miguez et al., 2010 (53)	2009	Retrospective (48 hrs post-partum)	245	Questionnaire	132 Drank once/week: 13 Once/2 weeks: 7 Once/month: 22 1-3 times/month: 89	53.9 Drank once/week: 5.3 Once/2 weeks: 2.9 Once/month: 9.0 1-3 times/month: 36.3	NA	NA

a Not available.

b Alcohol Use Disorders Identification Test.

c See https://pubs.niaaa.nih.gov/publications/arh28-2/78-79.htm for the specifics of these alcohol assessment questionnaires. Accessed on 10 April 2017. *Source:* Prepared by the authors from the study data.

### Meta-analyses

**FIGURE 1.**
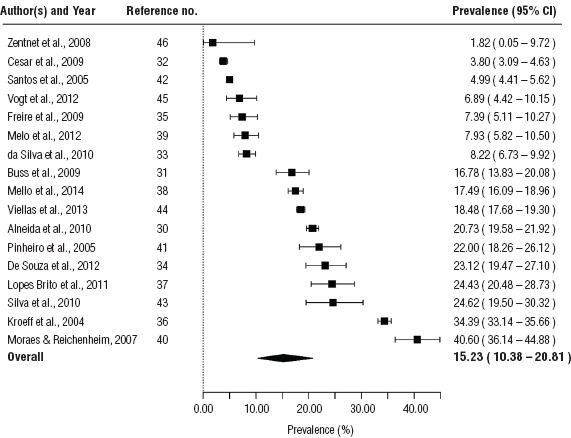
Forest plot of the prevalence estimates of alcohol consumption (any amount) during pregnancy among the general population of Brazil, 2004–2013

It was only possible to conduct a meta-analysis for Brazil and Mexico, based on the criterion of three available studies per country. The pooled prevalence of alcohol consumption during pregnancy among the general population in Brazil was estimated to be 15.2% (95%CI: 10.4%–20.8%), and contained estimates ranging from 1.8% (46) to 40.6% (40; Figure 1).

**FIGURE 2.**
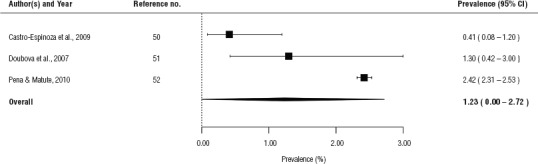
Forest plot of the prevalence estimates of alcohol consumption (any amount) during pregnancy among the general population of Mexico, 2007, 2009, 2010

***Source:*** Prepared by the authors from the study data.

The pooled prevalence of alcohol consumption during pregnancy among the general population in Mexico was estimated to be 1.2% (95%CI: 0.0% – 2.7%), and contained estimates ranging from 0.4% (50) to 2.4% (52; Figure 2).

The tests of heterogeneity demonstrated that heterogeneity was present in the estimates of alcohol consumption during pregnancy (I^2^ = 99.4% for Brazil and I^2^ = 89.1% for Mexico). We did not find evidence for the presence of publication bias in these meta-analyses (*P* = 0.326 for Brazil and *P* = 0.370 for Mexico).

### Data prediction

The prevalence of alcohol consumption during pregnancy was predicted for 31 countries with either no published data or fewer than three studies available. The three countries with the highest predicted prevalence of alcohol consumption during pregnancy among the general population were Grenada (23.3%, 95%CI: 20.1%–26.5%), St. Lucia (21.8%, 95%CI: 19.0%–24.7%), and Guyana (18.2%, 95%CI: 16.1%–20.2%; Table 2). The three countries with the lowest predicted prevalence of alcohol consumption during pregnancy among the general population were Cuba (4.8%, 95%CI: 4.2%–5.4%), Trinidad and Tobago (5.6%, 95%CI: 4.8%–6.5%), and Guatemala (6.5%, 95%CI: 5.6%–7.4%; Table 2).

**FIGURE 3.**
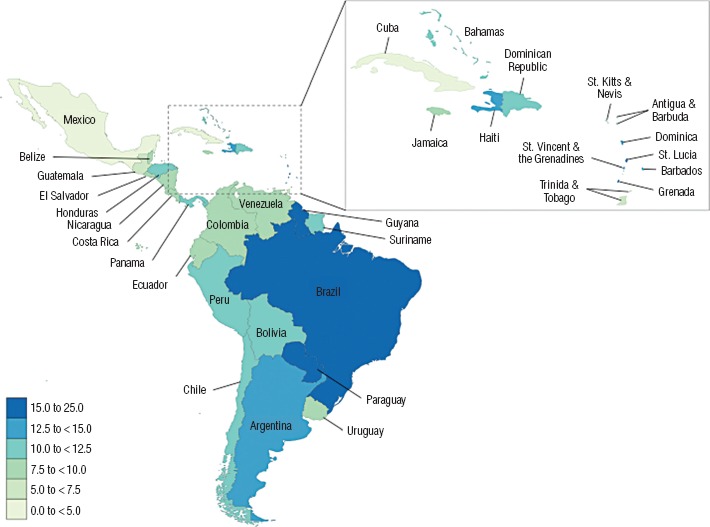
Prevalence of alcohol consumption during pregnancy among the general population of Latin America and the Caribbean, based on the two methods (meta-analyses versus weighted regression modelling/data prediction), 2012

***Note:*** Prediction of the prevalence of alcohol consumption during pregnancy for Argentina was not possible due to missing data.

***Source:*** Prepared by the authors from the study data.

The prevalence of alcohol consumption during pregnancy among the general population appears to be the highest in countries of the Caribbean and South America—61.5% of Caribbean countries and 75.0% of South American countries had a prevalence greater than or equal to 10%, compared to 25.0% in Central America (Figure 3).

**TABLE 2.** The prevalence of alcohol consumption (any amount) during pregnancy among the general population of Latin America and the Caribbean, by country, 2012

Country	Estimate (%)	95% Confidence Interval	
		Lower (%)	Upper (%)
Caribbean			
Antigua and Barbuda	9.7	8.3	11.0
Bahamas	12.0	10.1	14.0
Barbados	14.7	12.7	16.7
Cuba	4.8	4.2	5.4
Dominica	14.6	12.9	16.3
Dominican Republic	12.1	10.7	13.5
Grenada	23.3	20.1	26.5
Haiti	14.9	13.3	16.6
Jamaica	9.4	8.3	10.5
St. Kitts and Nevis	9.5	8.2	10.8
St. Lucia	21.8	19.0	24.7
St. Vincent and Grenadines	14.9	13.2	16.7
Trinidad and Tobago	5.6	4.8	6.5
Central America			
Belize	9.6	8.4	10.7
Costa Rica	8.3	7.3	9.3
El Salvador	8.3	7.3	9.3
Guatemala	6.5	5.6	7.4
Honduras	10.6	9.4	11.9
Mexico^a^	1.2	0.0	2.7
Nicaragua	8.9	7.8	10.0
Panama	11.7	10.1	13.2
South America			
Argentina	12.9	11.1	14.7
Bolivia	10.5	9.3	11.7
Brazil^a^	15.2	10.4	20.8
Chile	10.6	9.1	12.2
Colombia	9.1	8.0	10.1
Ecuador	8.9	7.8	9.9
Guyana	18.2	16.1	20.2
Paraguay	17.9	15.9	20.0
Peru	12.5	11.0	13.9
Suriname	10.2	8.9	11.4
Uruguay	8.8	7.6	9.9
Venezuela	10.0	8.7	11.2

a Estimate based on a meta-analysis of the current literature. ***Source:*** Prepared by the authors from the study data.

## DISCUSSION

The prevalence of alcohol consumption during pregnancy among the general population of the Caribbean ranged from 4.8% in Cuba to 23.3% in Grenada; in Central America, from 1.2% in Mexico to 11.7% in Panama; and in South America, from 8.8% in Uruguay to 18.2% in Guyana. Overall, our findings show a relatively high prevalence of alcohol consumption during pregnancy among the general population in some LAC countries, with 18 countries having an estimated prevalence of greater than or equal to 10%. Although some LAC countries have clinical guidelines that advise women to abstain from alcohol during pregnancy—Brazil (54, 55), Chile (56, 57), Cuba (58), and Mexico (59)—and may explain lower prevalence rates (in Cuba and Mexico), there is still an urgent need to educate both men and women of childbearing age on the detrimental consequences of consuming alcohol during pregnancy.

The high prevalence of alcohol consumption during pregnancy in some LAC countries could potentially be reflective of the pervasive alcohol marketing to women and the thriving alcohol industries that exist. For example, Chile is ranked among the top 10 wine-producing countries in the world, and several other South American countries have impressive beer-production industries, including Brazil, which ranks among the top five worldwide (60).

However, some of the identified studies report an unrealistically high prevalence of alcohol consumption during pregnancy among the general population. For example, the prevalence reported for Chile (57.4%; 47) and Uruguay (53.9%; 53) are notably higher than the estimates predicted for these countries (10.6%; 95%CI: 9.1%–12.2%, and 8.8%; 95%CI: 7.6%–9.9%, respectively). These reported prevalence rates also approach, and in the case of Uruguay exceed, the prevalence of current female drinkers (15+ years of age) among the general population in these countries (approximately 59% in Chile and 48% in Uruguay; 61). As such, the predicted prevalence of alcohol consumption during pregnancy is much more realistic for those countries with fewer than three available studies.

Furthermore, a recently published study from Argentina reported an alarmingly high prevalence, with 75% of women in the general population consuming at least one drink (10mL – 12mL) of alcohol during their pregnancy (62). However, according to Pan American Health Organization, only 48% of females in the general population of Argentina currently consume alcohol (61). Therefore, the predicted prevalence for Argentina (12.9%; 95%CI: 11.1%–14.7%) is more reasonable. Similarly, a study utilizing meconium testing in Montevideo, Uruguay, reported that 43.5% of samples tested positive for fatty acid ethyl esters above standard cut-off levels (i.e., 2 n mol/g) among women from low- and mid-to-low socioeconomic levels (63).

The comprehensive search strategy, *a priori* inclusion and exclusion criteria, and statistical analysis are notable strengths of the current study. Additionally, studies with sample composed of participants with low socioeconomic status or high-risk behaviors (e.g., all smokers) were excluded from the current study; thus, the generalizability of the findings to the general population is strengthened.

### Limitations

A few limitations should be noted. First, the majority of studies included in the current analysis utilized non-probability sampling and did not use validated tools to ascertain alcohol consumption during pregnancy. However, recent research shows that non-probability sampling techniques (e.g., convenience sampling) can be a suitable sampling strategy when exploring exposures during pregnancy (64). Also, it has been shown that a single question can detect as many (if not more) women who drink as other commonly used prenatal screens (65). Second, data on alcohol consumption during pregnancy were obtained through self-reported measures; therefore, reporting and recall biases may be present. As such, the prevalence of alcohol consumption during pregnancy may be underestimated in the current study. Third, the predicted prevalence values may differ from the true prevalence for a few reasons, namely: (i) data from which the values are predicted are not flawless, (ii) there may be other factors influencing the prevalence (e.g., different health and alcohol policies in different countries) that were not possible to take into account in the prediction model, and (iii) the ecological predictors may not allow for a precise estimate of the prevalence of alcohol consumption during pregnancy in the respective country. Despite these limitations, this study used the best available data and provides a working estimate of the prevalence of alcohol consumption during pregnancy in countries of LAC that do not currently have actual data. When data becomes available, further research can refine these estimates over time.

## Conclusions

Data on the prevalence of alcohol consumption during pregnancy in countries of Latin America are scarce, and in the Caribbean, are completely absent. As such, there is an urgent need for high quality studies to be conducted in countries of LAC. Regardless, this study’s findings point to the fact that measures are needed to deter pregnant women from consuming alcohol, and thus, reduce the occurrence of prenatal alcohol exposure and FASD. As prevention is key, educating women of childbearing age about the potential adverse consequences is of utmost importance.

When an infant with FASD is born, it is imperative that the mother receive substance abuse treatment. Treatment will reduce the chances of subsequent children with FASD, and increase the likelihood that the affected child and mother will be routinely monitored. Therefore, substance abuse treatment programs should be geared toward women of childbearing age with alcohol use disorders to prevent FASD occurrence/reoccurrence. Furthermore, alcohol marketing and promotion targeting female adolescents and women of childbearing age should be banned or strictly regulated by governments. Alcohol producers, distributors, and sellers should voluntarily discontinue such marketing, thereby potentially helping to reduce alcohol-related harm in children.

## Disclaimer.

Authors hold sole responsibility for the views expressed in the manuscript, which may not necessarily reflect the opinion or policy of the *RPSP/PAJPH* and/or PAHO.

## Footnotes

Central America: Belize, Costa Rica, El Salvador, Guatemala, Honduras, Mexico, Nicaragua, and Panama; South America: Argentina, Bolivia, Brazil, Chile, Colombia, Ecuador, Guyana, Paraguay, Peru, Suriname, Uruguay, and Venezuela; and the Caribbean: Antigua and Barbuda, Bahamas, Barbados, Cuba, Dominica, Dominican Republic, Grenada, Haiti, Jamaica, Saint Kitts and Nevis, Saint Lucia, Saint Vincent and the Grenadines, and Trinidad and Tobago.
